# Trends and interrupted time series analysis of congenital heart disease mortality among the Chinese population aged 0–64 years, 2002–2021

**DOI:** 10.1371/journal.pone.0354694

**Published:** 2026-08-10

**Authors:** Runfang Tian, Yucan Deng, Jinfeng Zhao, Li Liu, Panpan Sun, Zhiguang Ping

**Affiliations:** 1 College of Public Health, Zhengzhou University, Zhengzhou, Henan, China; 2 School of Basic Medical Sciences, Zhengzhou University, Zhengzhou, Henan, China; 3 Institute of Reproductive Health, Henan Academy of Innovations in Medical Science, National Health Commission Key Laboratory of Birth Defects Prevention, Zhengzhou, Henan, China; 4 Department of Epidemiology and Biostatistics, School of Public Health, Xi’an Jiaotong University Health Science Center, Xi’an, Shaanxi, China; GH Raisoni College of Engineering and Management Pune, INDIA

## Abstract

**Objectives:**

Our study aimed to analyze congenital heart disease (CHD) mortality trends among Chinese residents aged 0–64 years from 2002 to 2021 and assess the impact of implementing the medical insurance policy (MIP) for rural children on CHD mortality.

**Methods:**

Data on CHD mortality in urban and rural regions of China from 2002 and 2021 were collected from the China Health Statistics Yearbook. Because mortality data for individuals aged ≥65 years were incomplete and lacked specificity for CHD, the analysis was restricted to those aged 0–64 years. Joinpoint regression was used to analyze trends in CHD mortality by region, sex, and age group, and interrupted time series analysis was used to evaluate the impact of the MIP on CHD mortality.

**Results:**

Between 2002 and 2021, the standardized CHD mortality among Chinese residents was higher in rural areas than in urban areas, and higher in males than in females. CHD mortality for rural and urban residents declined at an average annual percentage change (AAPC) of −3.63% (95% CI: −4.84% ~ −2.40%) and −4.99% (95% CI: −8.38% ~ −1.47%), respectively. Male mortality decreased more than female mortality. Mortality in the < 1, 1−4, 5−9, 35−39, and 40−44 age groups have been declining over the past 20 years; Furthermore, CHD mortality in rural areas has gradually decreased since the implementation of MIP (*β*_*3*_ = −0.073,95% CI: −0.100 ~ −0.045).

**Conclusions:**

In China, CHD mortality declined from 2002 to 2021, with variations still present based on gender, age, and region. Future prevention and control measures should be strengthened for specific populations in different regions.

## 1. Background

Congenital heart disease (CHD) is a common developmental structural defect of the cardiovascular system and is usually present at birth. CHD tops the list of neonatal congenital disabilities in many parts of the country. The detection rate continues to rise among newborns nationwide, and it is projected that there are 2 million patients with CHD in China [1]. CHD is one of the leading causes of death in children under the age of 5 years [2], and the lack of diagnosis and treatment leads to the death of approximately one-third of the patients in the first year of life [3]. CHD and its complications place a heavy financial burden on families and society, and its treatment costs are the highest among neonatal patients [4].

The trend of disease mortality is the most intuitive measure of a country’s disease burden [5]. Our study analyzed the trend of CHD mortality in China from 2002 to 2021, aiming to gain a comprehensive understanding of the trend of CHD mortality in different regions, genders, and age groups and the possible influencing factors. In particular, a medical insurance policy (MIP) for rural children was launched in 2010 to cover approximately 70–90% of surgical costs for CHD, potentially reducing mortality in rural areas. To assess both the overall trend and the policy impact, we employed two complementary methods: Joinpoint regression, which describes long-term trends and identifies natural breakpoints without prespecifying an intervention, and interrupted time series analysis (ITSA), which evaluates the causal effect of the 2010 MIP by estimating immediate level changes and slope changes after the policy. This will help us better explore the epidemiological characteristics of CHD in China and provide a scientific basis for future disease prevention and treatment.

## 2. Methods

### 2.1. Data sources

Data on CHD mortality for rural and urban populations were obtained from the China Health Statistics Yearbook 2003−2022 [6] (hereinafter referred to as the Yearbook), published by the Statistical Information Center of the National Health Commission. The Yearbook’s data are compiled from annual mortality reports submitted by health authorities at multiple administrative levels, with all diagnoses standardized to the ICD-10. We analyzed CHD mortality data across a 20-year period (2002–2021), with stratification by urban-rural residence, sex, and age. As this study used publicly available secondary data, ethical approval was not applicable.

### 2.2. Grouping of the study population

In the Yearbook, ages are classified as <1 year, 1–4 years, 5–9 years, 10–14 years,......, 80–84 years, 85 years and over. However, Mortality rates for CHD were low among those aged ≥65 years, and deaths in this age group may involve multiple chronic conditions rather than CHD alone. Additionally, data were incomplete for this age cohort. To ensure reliable findings, we restricted the analysis to the 0–64 years age group.

### 2.3. Statistical analysis

#### 2.3.1. Standardization of CHD mortality.

The CHD mortality data from 2002–2021 was compiled with Excel 2019, the data from the Seventh National Population Census in 2020 was used as the standard population, adjusted for age and sex, respectively, to remove the impacts of demographic shifts. Meanwhile, as the CHD mortality in towns and villages were not reported separately in the Yearbook, our study referred to the delineation criteria of previous study [7], using the census urban population as the urban standard population and combining towns and villages as the rural population for the standardization process, finally, we calculated the overall urban and rural standardized mortality, as well as the urban and rural standardized mortality for CHD by sex and age, respectively. All data in our study are using standardized mortality(1/100,000) (conveniently referred to as mortality). Details of the standardization process are provided in the Supplementary Material.

#### 2.3.2. Joinpoint regression analysis.

Joinpoint regression analysis can identify the presence of joinpoints in the data and fit a trendline to each trending segment. In our study, a Joinpoint regression model was built with Joinpoint 5.1.0.0 to evaluate the trends in mortality of patients in different regions, sexes, and age groups by calculating annual percent change (APC) and average annual percent change (AAPC). APC > 0 or AAPC>0 indicated that the mortality of CHD increased over time, and vice versa; APC = AAPC indicates that no joinpoints were identified, and the general trend remained unchanged [8]. The significance level *α* of both sides is taken as 0.05.

#### 2.3.3. Interrupted time series analysis (ITSA).

Interrupted time series analysis is the most commonly used quasi-experimental design for assessing the impact of interventions, which collects data related to outcome variables measured at multiple time points before and after the implementation of the intervention and evaluates the effectiveness of the intervention by analyzing changes before and after the intervention [9].

The medical insurance policy evaluated in this study was officially launched in 2010, first piloted in some provinces, and then extended to all provinces in China in 2011. The policy reduced the financial burden on families by covering approximately 70% of surgical costs through the New Rural Cooperative Medical Scheme (NRCMS), with an additional 20% covered by medical financial assistance, thereby capping household out-of-pocket expenses at about 10% [10].This approach reduces financial barriers and enhances healthcare accessibility, enabling patients with mild conditions to receive timely treatment, while those with severe cases may also experience prolonged survival [11]. An interrupted time series analysis was conducted using Stata 16.0 to evaluate the impact of the medical insurance policy (MIP) on CHD mortality. Given that mortality rates are bounded, natural logarithmic transformation (ln*Y*) was applied to the age-standardized CHD mortality rates prior to modeling. The model was as follows:


$$\begin{array}{c} \text{ln}Y=\beta_{\text{0}}+\beta_{\text{1}}\times \text{time}+\beta_{\text{2}}\times \text{intervention}+\beta_{\text{3}}\times \text{time}\_\text{after}\_\text{intervention}+\varepsilon \end{array}$$

(‘*Y*’ represents the mortality associated with CHD; ‘time’ is a time variable expressed in years from the observation period; ‘intervention’ denotes the implementation or non-implementation of the policy; and ‘time_after_intervention’ is the time series following the intervention; *β*_*0*_,*β*_*1*_,*β*_*2*_ and *β*_*3*_ are model parameters, where *β*_*0*_ is a constant term, and *β*_*1*_ is the slope before the policy; *β*_*2*_ is the amount of immediate level change, reflecting the magnitude of the change in the immediate level of the mortality of CHD before and after the policy; *β*_*3*_ is the amount of change in the slope after the policy, with (*β*_*1*_ + *β*_*3*_) being the slope after the policy; ε is the error.)

The short-term impact and long-term trend of implementing the MIP on CHD mortality were determined by estimates of *β*_*2*_ and *β*_*3*_. Given the small sample size (n = 20), the Durbin-Watson test had limited statistical power to detect autocorrelation, and heteroscedasticity was not considered. Therefore, we used the Newey-West estimator to calculate the standard error (lag order = 2). Furthermore, given the reasonable delay from pilot implementation to full coverage and from surgical intervention to mortality reduction, a sensitivity analysis was conducted, and the model was refitted, assuming that the policy was implemented in 2011 and 2012 respectively.

## 3. Results

### 3.1. Changes in CHD mortality over 20 years

**Fig 1:**
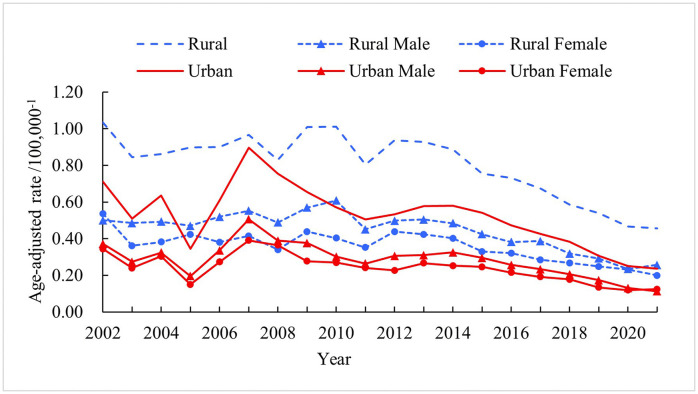
Changes in standardized CHD mortality by sex in urban and rural (1/100,000).

As shown in Fig 1, during the period 2002–2021, both rural and urban regions showed a fluctuating decline in the mortality for CHD. Overall, rural mortality was higher than those in urban regions. Regarding sex and region, rural males had the highest mortality, followed by rural females, urban males, and urban females.

### 3.2. Analysis of changing trends in CHD mortality

#### 3.2.1. Comparison of trends in CHD mortality for urban and rural residents.

**Fig 2:**
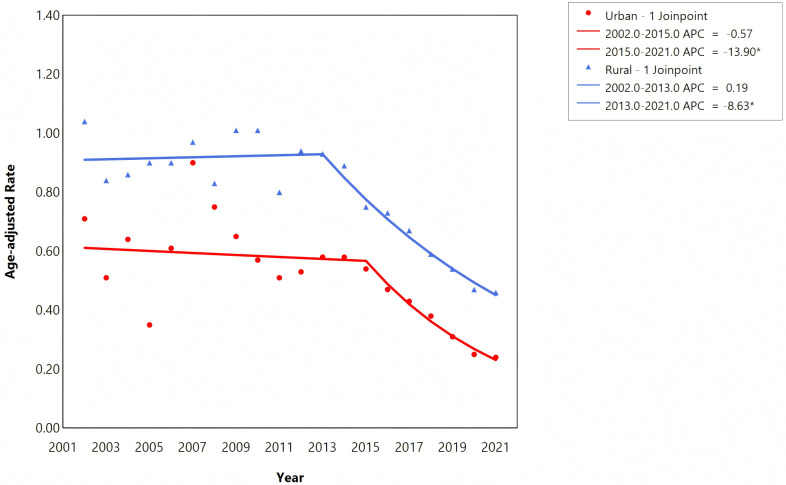
Trends in CHD mortality for urban and rural residents, 2002-2021.

As shown in Table 1 and Fig 2, during 2002 and 2021, the CHD mortality decreased for both rural and urban (rural: AAPC = −3.63%, 95% CI: −4.84% ~ −2.40%; urban: AAPC = −4.99%, 95% CI: −8.38% ~ −1.47%). Both rural and urban regions had one joinpoints, with the former(joinpoints:2013) having joinpoints before the latter (joinpoints:2015): In rural regions, the trend was not statistically significant (*P* > 0.05) from 2002–2013 and showed a rapid decline in 2013–2021 (APC = −8.63%, 95% CI: −10.89% ~ −6.33%); in urban regions, the trend was not statistically significant (*P* > 0.05) from 2002 to 2015 and declined significantly in 2015–2021, with an APC as high as −13.90% (95% CI: −22.40% ~ −4.47%).

**Table 1:** Temporal trends in CHD mortality for urban and rural residents, 2002-2021.

Region	Year	APC(95%*CI*)%	*t/P*	AAPC(95%*CI*)%	*t/P*
Rural	2002—2013	0.19(−1.35 ~ 1.74)	0.26/0.799	−3.63(−4.84 ~ −2.40)	−5.71/ < 0.001
	2013—2021	−8.63(−10.89 ~ −6.33)	−7.72/ < 0.001		
Urban	2002—2015	−0.57(−3.73 ~ 2.68)	−0.38/0.709	−4.99(−8.38 ~ −1.47)	−2.76/0.006
	2015—2021	−13.90(−22.40 ~ −4.47)	−3.07/0.008		

Abbreviations: AAPC, average annual percentage change; APC, annual percentage change.

#### 3.2.2. Comparison of trends in CHD mortality by sex.

In both rural and urban regions, the decline in mortality was greater for males (rural: AAPC = −3.72%, 95% CI: −5.04% ~ −2.38%; urban: AAPC = −5.35%, 95% CI: −8.72% ~ −1.86%) than for females (rural: AAPC = −3.70%, 95% CI: −5.46% ~ − 1.91%; urban: AAPC = −4.83%, 95% CI: −8.45% ~ −1.07%).

**Fig 3:**
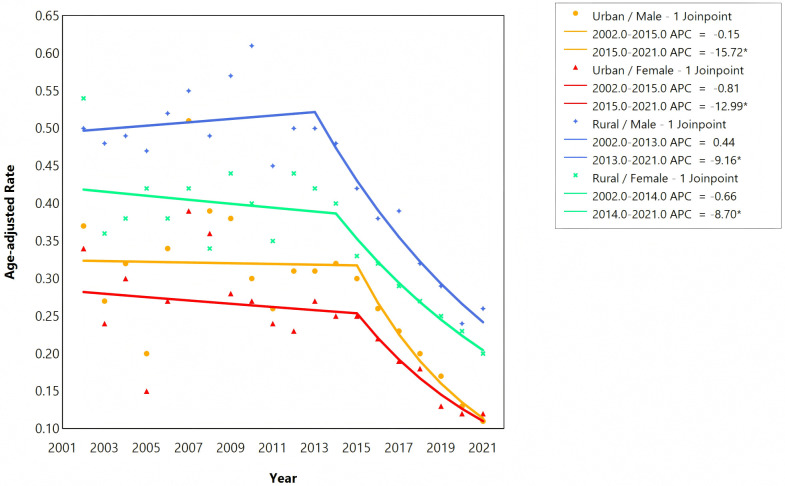
Trends in CHD mortality for urban and rural residents by sex, 2002-2021.

As shown in Table 2 and Fig 3, for rural residents, the trend change was not statistically significant for males and females during 2002−2013 and 2002−2014 (P > 0.05), respectively, and decreased after 2013 and 2014 at a rate of 9.16% (95% CI: −11.60% ~ −6.66%) and 8.70% (95% CI: −12.58% ~ −4.65%). For urban residents, there was no statistically significant trend in mortality change during 2002−2015 (*P* > 0.05) and a significant decline during 2015−2021 (male: APC = −15.72%, 95% CI: −24.00% ~ −6.53%; female: APC = −12.99%, 95% CI: −22.11% ~ −2.81%).

**Table 2:** Analysis of time trends in CHD mortality for urban and rural residents by sex, 2002-2021.

Region	Year	APC(95%*CI*)%	*t/P*	AAPC(95%*CI*)%	*t/P*
Rural Male	2002—2013	0.44(−1.23 ~ 2.14)	0.56/0.582	−3.72(−5.04 ~ −2.38)	−5.39/ < 0.001
	2013—2021	−9.16(−11.60 ~ −6.66)	−7.55/ < 0.001		
Rural Female	2002—2014	−0.66(−2.55 ~ 1.27)	−0.73/0.476	−3.70(−5.46 ~ −1.91)	−4.00/ < 0.001
	2014—2021	−8.70(−12.58 ~ −4.65)	−4.47/ < 0.001		
Urban Male	2002—2015	−0.15(−3.30 ~ 3.11)	−0.10/0.922	−5.35(−8.72 ~ −1.86)	−2.98/0.003
	2015—2021	−15.72(−24.00 ~ −6.53)	−3.52/0.003		
Urban Female	2002—2015	−0.81(−4.16 ~ 2.65)	−0.50/0.621	−4.83(−8.45 ~ −1.07)	−2.51/0.012
	2015—2021	−12.99(−22.11 ~ −2.81)	−2.68/0.017		

#### 3.2.3. Trend analysis of CHD mortality for different age groups.

The mortality for the following age groups decreased between 2002 and 2021: < 1 year, 1–4 years, 5–9 years, 35–39 years, and 40–44 years. These declines occurred at an average annual rate of 6.49%, 5.44%, 3.71%, 2.99%, and 2.25%, respectively (*P* < 0.05). The other age groups did not have statistically significant AAPC values between 2002 and 2021 (*P* > 0.05). Furthermore, the 15–19 and 20–24 age groups experienced declines of 2.21% and 7.46% in 2005–2021 and 2008–2018, respectively (P < 0.05); the 25–29 and 60–64 age groups experienced increases of 3.63% and 22.99% in 2002–2016 and 2002–2009, respectively (P < 0.05). Refer to Table 3.

**Table 3:** Analysis of temporal trends in CHD mortality in different age groups, 2002-2021.

Age group	Year	APC(%)	*P*	AAPC(%)	*P*
<1	2002—2014	0.05	0.977	−6.49	<0.001
	2014—2021	−16.72	< 0.001		
1 ~ 4	2002—2010	5.11	0.052	−5.44	< 0.001
	2010—2021	−12.44	< 0.001		
5 ~ 9	2002—2021	−3.71	< 0.001	−3.71	< 0.001
10 ~ 14	2002—2021	1.38	0.237	1.38	0.237
15 ~ 19	2002—2005	21.39	0.100	1.19	0.531
	2005—2021	−2.21	0.019		
20 ~ 24	2002—2008	7.15	0.070	−0.06	0.977
	2008—2018	−7.46	0.001		
	2018—2021	12.32	0.280		
25 ~ 29	2002—2016	3.63	0.023	−0.22	0.916
	2016—2021	−10.24	0.127		
30 ~ 34	2002—2005	−17.82	0.249	−1.19	0.668
	2005—2021	2.28	0.092		
35 ~ 39	2002—2006	−10.14	0.044	−2.99	0.008
	2006—2021	−0.98	0.149		
40 ~ 44	2002—2021	−2.25	0.002	−2.25	0.002
45 ~ 49	2002—2021	1.12	0.231	1.12	0.231
50 ~ 54	2002—2021	1.58	0.401	1.58	0.401
55 ~ 59	2002—2021	−0.04	0.981	−0.04	0.981
60 ~ 64	2002—2009	22.99	0.041	4.33	0.321
	2009—2021	−5.22	0.210		

### 3.3. Effects of MIP implementation on CHD mortality in rural regions

**Fig 4:**
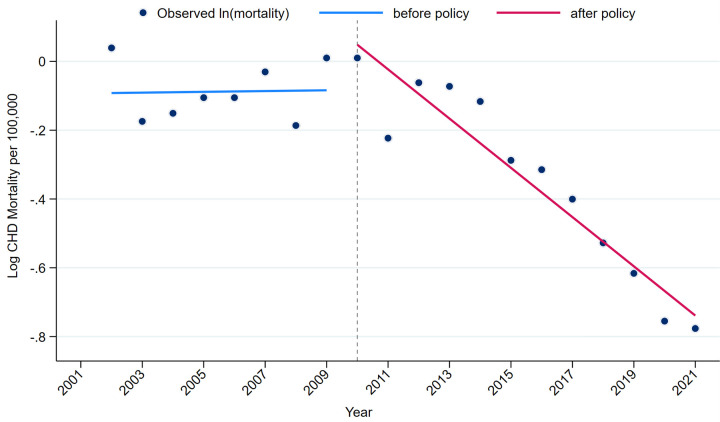
Interrupted time series of CHD mortality levels for the rural residents, 2002-2021.

China launched the MIP for rural children in 2010, and its impact on CHD mortality was investigated in this study with ITAS. The interrupted time series analysis revealed no significant pre-policy time trend in log-transformed rural CHD mortality (*β*_*1*_ = 0.001, *P* = 0.923) and no significant immediate level change in 2010 (*β*_*2*_ = 0.131, *P* = 0.20). However, a significant post-policy slope change was observed (*β*_*3*_ = −0.073, *P* < 0.001), indicating an accelerated long-term decline in rural CHD mortality after the implementation of the MIP. Model diagnostics confirmed no significant residual autocorrelation up to order 2 (Breusch-Godfrey LM test: lag 1 *P* = 0.895, lag 2 *P* = 0.781) and no evidence of heteroskedasticity on the log scale (Breusch-Pagan test, *P* = 0.195). Newey-West heteroskedasticity- and autocorrelation-consistent (HAC) standard errors with a maximum lag of 2 were used to ensure robust inference. Refer to Fig 4 and Table 4.

**Table 4:** Results of interrupted time series analysis for rural CHD mortality(2010).

	*β*	Newey–West *SE*	*t*	*P*	95%*CI*
*β*_*0*_	−0.092	0.053	−1.73	0.102	(−0.204 ~ 0.020)
time(*β*_*1*_)	0.001	0.012	0.10	0.923	(−0.024 ~ 0.026)
intervention(*β*_*2*_)	0.131	0.100	1.32	0.205	(−0.079 ~ 0.342)
time_after_intervention(*β*_*3*_)	−0.073	0.013	−5.59	<0.001	(−0.100 ~ −0.045)

Model diagnostics for the rural ITS model (log scale): Breusch–Godfrey LM test for autocorrelation up to order 2: lag 1 P = 0.895, lag 2 P = 0.781; Breusch–Pagan test for heteroskedasticity: P = 0.195. Newey–West HAC standard errors with lag 2 are reported to ensure robust inference.

To account for potential lagged policy effects, the intervention onset was shifted to 2011 and 2012, respectively. In the one-year lag model, the post-policy slope change remained significantly negative (*β*_*3*_ = −0.081, *P* < 0.001). In the two-year lag model, the slope change was also significant (*β*_*3*_ = −0.085, *P* < 0.001), with a positive immediate level change (*β*_*2*_ = 0.109,*P* = 0.038), likely reflecting improved case ascertainment as policy coverage expanded. Overall, the sensitivity analyses corroborate the main finding of an accelerated long-term decline in rural CHD mortality following policy implementation. Refer to S4 Table and S5 Table in S1 File.

## 4. Discussion

Based on Yearbook data, our study systematically analyzed trends in CHD mortality for patients of various age groups, sexes, and regions in China from 2002 to 2021. We also employed interrupted time series analyses to examine the effects of the MIP implementation on CHD mortality. The findings demonstrated a declining trend in CHD mortality among Chinese residents from 2002 to 2021, with a larger decline in the mortality of males than females in both rural and urban regions. The trend of mortality varied by age group. In addition, the CHD mortality has fallen significantly following the implementation of MIP. The findings of this research can serve as a useful guide for future efforts to prevent and treat CHD.

China has made great progress in the prevention and treatment of CHD, but still faces the threat of high mortality. The mortality trend among Chinese individuals with CHD from 2002 to 2021 exhibited an overall decrease, aligning with related studies [11]. However, the prevalence and mortality of CHD in China still face challenges. A study examining global, regional, and national trends in CHD mortality revealed that China’s all-age mortality rate remains 2.5 to 4.5 times greater than that of countries with a high socio-demographic index (SDI) [12]. Another study found that the mortality of lower-age patients in China is still higher than that in North America [13]. These indicate that despite progress in the prevention and treatment of CHD in China, it remains a significant health threat compared to some developed nations and regions. Consequently, China must enhance its strategies for preventing, diagnosing, and treating CHD to decrease its mortality further.

There are regional differences in the changing trend of CHD mortality rates. CHD mortality was higher among rural residents than urban residents, consistent with previous studies [2]. This implies that the situation for CHD mortality is more dire in rural regions, and it is crucial to consider how socioeconomic variables affect CHD deaths [14]. In addition, urban and rural mortality rates decreased substantially in 2015–2021 and 2013–2021, respectively, which may be related to the following factors: Firstly, in 2010, China’s Ministry of Health initiated a pilot program to safeguard rural children against CHD [10], significantly alleviating the financial burden on rural residents; Secondly, in the past 10 years, with the development of pediatric cardiac surgery and the continuous improvement of the success rate of surgery, the cure rate of CHD has been significantly improved [15]; Thirdly, screening technologies such as echocardiography are constantly evolving [16]. In 2018, the National Health and Health Commission included neonatal CHD screening in the neonatal disease screening spectrum [17] to facilitate early detection of CHD, so as to reduce the mortality of neonatal and infant children. Additionally, urban regions outperform rural regions in terms of economic and health conditions, as well as the acceptance and implementation of health therapies, resulting in temporal differences in the joinpoints of CHD mortality between the two regions.

There are sex differences in the changing trend of CHD mortality. Our study found that CHD mortality was overall higher in males than in females, both in urban and rural regions, suggesting that the risk of death from CHD is higher in males than in females. This discrepancy may be attributed to a multifactorial etiology. Anatomically, male neonates tend to present with more complex congenital heart defects, in contrast to females, who more commonly display non-cardiac structural abnormalities and lower-risk cardiac pathologies necessitating less complex surgical care [18–20]. This early-life divergence persists into adulthood, where nearly 20% of patients require cardiovascular surgery within 15 years. Males are not only more frequently indicated for such procedures but also show inferior long-term survival [21]. These findings collectively suggest that males face a relatively disadvantageous position from the initial severity of the condition through to subsequent treatment burdens. Furthermore, gender differences in risk factors and health behaviors also contribute to outcome disparities. Males are more adversely affected by dyslipidemia and depression and typically engage in fewer health-promoting behaviors—such as healthy eating, smoking cessation, and regular exercise—than females [22,23].Currently, the underlying mechanisms remain poorly elucidated; future studies should examine the roles of genetic predisposition, hormonal influences, behavioral patterns, and other unidentified factors in driving these sex-based differences [24,25].

There are age-specific differences in the changes in CHD mortality. According to the Joinpoint analysis of CHD mortality by age, children in China aged 0–4 years experienced the largest decline between 2002 and 2021, which was in line with the global trend [12]. The age group under 1 year experienced the fastest decline, which could be attributed to advancements in screening technology, maternal testing awareness, and treatment quality. It should be noted, however, that CHD remains one of the leading causes of death for children under five in China [26,27]. A related study revealed that the percentage of CHD deaths among children under one year old has been rising annually [28]. This could be because the mortality of other diseases among children under one year old is declining more quickly than CHD mortality, which leads to an increase in the proportion of deaths caused by CHD. Thus, we should promote prenatal screening and bolster health education during pregnancy to allow prompt detection and early treatment. Furthermore, CHD mortality in the 15–19 and 20–24 age groups exhibited a declining trend from 2005 to 2021 and 2008–2018, respectively, this trend may be attributed to advancements in early diagnostic methodologies and therapeutic interventions that enable physicians to address the disease at earlier stages. On the other hand, between 2002 and 2016, the CHD mortality in the 25–29 age group increased. This may be related to the fact that people in this age group have just entered the workforce, face greater work pressure, and have unhealthy lifestyle choices. In addition, CHD mortality among individuals aged 35–44 demonstrated a declining trend over the 20 years, possibly as a result of their strong financial standing and long-standing experience with cardiac self-care, which enables them to take long-term control of their health. In conclusion, focused preventative and control interventions across various age groups are crucial for diminishing the overall mortality of CHD across the life cycle.

Our study examined the impact of the MIP for rural children on CHD mortality. Interrupted time series analysis showed that the immediate level change following policy implementation was not statistically significant, whereas the long-term trend change was significant. This pattern likely reflects regional variations in policy intensity and timing, given the initial pilot in select provinces (2010) followed by nationwide expansion (2011). Sensitivity analyses shifting the intervention onset to 2011 and 2012 consistently yielded significantly negative long-term slope changes. Notably, the two-year lag model showed a statistically significant positive immediate level change, which probably reflects improved case ascertainment and more complete mortality registration under full coverage rather than a true increase in CHD burden. The robustness of the accelerated long-term decline across different intervention start points supports the conclusion that the MIP contributed to a sustained reduction in rural CHD mortality, despite the absence of an immediate level shift in the primary analysis. It is important to note, however, that the observed post-policy acceleration in rural CHD mortality cannot be attributed solely to the MIP. During the same period, several concurrent factors likely contributed, including the continuous improvements in pediatric cardiac surgery, the national rollout of neonatal CHD screening, and broader socioeconomic development. These factors may have acted synergistically with the MIP to reduce mortality.In conclusion, the MIP meaningfully accelerated the long-term decline in rural CHD mortality, demonstrating that targeted health insurance for surgically correctable pediatric conditions can yield sustained mortality benefits even without an immediate level shift. This finding supports extending such policies to other high-burden, surgically treatable diseases in low-resource settings.

In light of the substantial decrease in the mortality of CHD, it is imperative to investigate the contributing factors of CHD. Beyond demographic variables—including region, gender, and temporal context—CHD arises from a multifactorial etiology. Genetically, mutations in approximately 400 genes are implicated in human CHD [29]. From an environmental perspective, maternal conditions such as diabetes and obesity during early pregnancy, as well as exposure to pollutants, have been shown to significantly increase the risk of cardiac malformations [30–32]. Socioeconomic and behavioral determinants, including low parental socioeconomic status and maternal smoking, are also associated with elevated CHD incidence. Collectively, these factors constitute a complex etiological network for CHD, underscoring the necessity for multifaceted prevention and control strategies [33,34]. Meanwhile, in response to the population with a high CHD mortality, it is imperative to implement a comprehensive CHD medical system to facilitate the ongoing reduction of CHD mortality.

Our study has the following advantages: 1. Based on the data from the Yearbook, the information source is reliable, and due to the long data collection period, the study’s conclusions were guaranteed to be accurate and dependable; 2. Our study updated the temporal trend in mortality due to CHD in China from 2002 to 2021, which spans a long period. 3. The study used two methods: the Joinpoint regression model and interruption time series analysis. The Joinpoint regression model can evaluate the changing trend of CHD mortality, while interrupted time series analysis can effectively identify the changes before and after policy intervention. These two methods complement each other and help comprehensively understand CHD’s changing characteristics in China in the past 20 years. Our study has several limitations. First, due to incomplete mortality data for CHD among individuals aged ≥65 years and poor cause-of-death specificity for CHD in the elderly, our analysis was restricted to the population aged 0–64 years; consequently. Second, the available data did not allow stratification by specific CHD subtypes nor analysis at the individual level, which prevented us from exploring risk factors associated with CHD mortality in greater depth. Third, the ITSA for the MIP lacked a contemporaneous control group and could not fully adjust for post‑2010 confounders, so residual confounding cannot be excluded. Future studies should collect more complete mortality data for the elderly, integrate diverse data sources to enable CHD subtype and individual‑level risk factor analysis, and employ region‑ or individual‑level data with appropriate control groups to more precisely quantify the causal effect of the MIP.

## 5. Conclusion

China’s efforts to prevent and control CHD have shown great success, leading to a large decline in CHD mortality. However, the mortality of CHD is still higher in rural regions than in urban regions, higher in males than in females in the same region, and there are differences in the mortality trends of different age groups. In the future, preventive and control measures should be strengthened for specific populations in different regions so as to reduce China’s CHD mortality further.

## Supporting information

S1 File S1-S5 Tables. (DOCX)
