# Supplementary material for: Trends and interrupted time series analysis of congenital heart disease mortality among the Chinese population aged 0–64 years, 2002–2021
Source: PLoS One. 2026 Aug 10;21(8):e0354694. doi: 10.1371/journal.pone.0354694 (PMC13456267; doi:10.1371/journal.pone.0354694)
Supplement: Supplementary file 1 [file pone.0354694.s001.docx]

**Supplementary Materials**

**Trends and interrupted time series analysis of congenital heart disease mortality in China from 2002 to 2021**

- Standardized processes for CHD mortality

According to the data of the seventh national population census in 2020, The standard population of urban and rural areas and gender were obtained according to the following age groups (< 1, 1-4, 5-9, 10-14, 15-19, 20-24, 25-29, 30-34, 35-39, 40-44, 45-49, 50-54, 55-59, 60-64). Among them, the rural standard population was calculated by combining the town population and the village population. Specific data are shown in S1 and S2.

**Table S1 Standard population in urban and rural areas**

|  | Urban | Rural |
| --- | --- | --- |
| ＜1 | 4497029 | 7491028 |
| 1-4 | 26104508 | 39791323 |
| 5-9 | 32260730 | 57983326 |
| 10-14 | 27347422 | 57908572 |
| 15-19 | 32654045 | 40030095 |
| 20-24 | 38258137 | 36683538 |
| 25-29 | 44415879 | 47431453 |
| 30-34 | 60075584 | 64069606 |
| 35-39 | 48129615 | 50883317 |
| 40-44 | 42221092 | 50734238 |
| 45-49 | 47056463 | 67168424 |
| 50-54 | 44885021 | 76279275 |
| 55-59 | 37888257 | 63512529 |
| 60-64 | 27440811 | 45942127 |
| $N$ | 1219143444 | |

**Table S2 Standard population by sex and region**

|  | Male（Urban） | Male（Rural） | Female（Urban） | Female（Rural） |
| --- | --- | --- | --- | --- |
| ＜1 | 2361683 | 3950726 | 2135346 | 3540302 |
| 1-4 | 13716841 | 20940081 | 12387667 | 18851242 |
| 5-9 | 17172999 | 30844459 | 15087731 | 27138867 |
| 10-14 | 14619691 | 30987099 | 12727731 | 26921473 |
| 15-19 | 17249362 | 21803981 | 15404683 | 18226114 |
| 20-24 | 19776472 | 19899523 | 18481665 | 16784015 |
| 25-29 | 22937131 | 25225139 | 21478748 | 22206314 |
| 30-34 | 30519381 | 33352427 | 29556203 | 30717179 |
| 35-39 | 24437714 | 26494323 | 23691901 | 24388994 |
| 40-44 | 21424853 | 26207841 | 20796239 | 24526397 |
| 45-49 | 23879328 | 34312358 | 23177135 | 32856066 |
| 50-54 | 22560304 | 38545166 | 22324717 | 37734109 |
| 55-59 | 18844970 | 31971056 | 19043287 | 31541473 |
| 60-64 | 13477770 | 23393355 | 13963041 | 22548772 |
| $N$ | 1219143444 | | | |

Based on the standard population obtained, the standardized mortality rate was calculated using the direct standardization method. The calculation formula is as follows:

$$P'=\frac{\sum N_{i}P_{i}}{N}$$

Among them, $P'$ is the standardized mortality rate, $P_{i}$ is the actual mortality rate of the ith age group, $N_{i}$ is the standard number of the ith age group in the standard group, and $N$ is the total standard population. The crude and standardized mortality by urban and rural areas and by sex are shown in S3.

**Table S3 Mortality rate of CHD in urban and rural from 2002 to 2021**

|  | Urban/(1/100,000) | | | | | | Rural/(1/100,000) | | | | | |
| --- | --- | --- | --- | --- | --- | --- | --- | --- | --- | --- | --- | --- |
|  | Male | | Female | | Sum | | Male | | Female | | Sum | |
| Year | CDR | SM | CDR | SM | CDR | SM | CDR | SM | CDR | SM | CDR | SM |
| 2002 | 2.06 | 0.37 | 1.74 | 0.34 | 1.91 | 0.71 | 1.77 | 0.50 | 2.01 | 0.54 | 1.89 | 1.04 |
| 2003 | 1.43 | 0.27 | 1.34 | 0.24 | 1.40 | 0.51 | 1.76 | 0.48 | 1.43 | 0.36 | 1.60 | 0.84 |
| 2004 | 1.26 | 0.32 | 1.25 | 0.30 | 1.26 | 0.64 | 1.55 | 0.49 | 1.31 | 0.38 | 1.43 | 0.86 |
| 2005 | 1.27 | 0.20 | 0.92 | 0.15 | 1.10 | 0.35 | 1.47 | 0.47 | 1.49 | 0.42 | 1.48 | 0.90 |
| 2006 | 1.39 | 0.34 | 1.16 | 0.27 | 1.28 | 0.61 | 1.64 | 0.52 | 1.26 | 0.38 | 1.45 | 0.90 |
| 2007 | 1.74 | 0.51 | 1.49 | 0.39 | 1.62 | 0.90 | 1.66 | 0.55 | 1.34 | 0.42 | 1.51 | 0.97 |
| 2008 | 1.51 | 0.39 | 1.53 | 0.36 | 1.52 | 0.75 | 1.58 | 0.49 | 1.14 | 0.34 | 1.36 | 0.83 |
| 2009 | 1.60 | 0.38 | 1.30 | 0.28 | 1.45 | 0.65 | 1.81 | 0.57 | 1.46 | 0.44 | 1.64 | 1.01 |
| 2010 | 1.36 | 0.30 | 1.26 | 0.27 | 1.31 | 0.57 | 1.77 | 0.61 | 1.30 | 0.40 | 1.54 | 1.01 |
| 2011 | 1.14 | 0.26 | 1.09 | 0.24 | 1.12 | 0.51 | 1.55 | 0.45 | 1.24 | 0.35 | 1.40 | 0.80 |
| 2012 | 1.36 | 0.31 | 1.07 | 0.23 | 1.21 | 0.53 | 1.70 | 0.50 | 1.50 | 0.44 | 1.60 | 0.94 |
| 2013 | 1.33 | 0.31 | 1.16 | 0.27 | 1.24 | 0.58 | 1.71 | 0.50 | 1.44 | 0.42 | 1.58 | 0.93 |
| 2014 | 1.38 | 0.32 | 1.10 | 0.25 | 1.24 | 0.58 | 1.71 | 0.48 | 1.39 | 0.40 | 1.55 | 0.89 |
| 2015 | 1.27 | 0.30 | 1.06 | 0.25 | 1.17 | 0.54 | 1.47 | 0.42 | 1.15 | 0.33 | 1.31 | 0.75 |
| 2016 | 1.11 | 0.26 | 0.93 | 0.22 | 1.02 | 0.47 | 1.32 | 0.38 | 1.13 | 0.32 | 1.02 | 0.73 |
| 2017 | 1.00 | 0.23 | 0.85 | 0.19 | 0.92 | 0.43 | 1.34 | 0.39 | 1.00 | 0.29 | 1.17 | 0.67 |
| 2018 | 0.88 | 0.20 | 0.80 | 0.18 | 0.84 | 0.38 | 1.10 | 0.32 | 0.93 | 0.27 | 1.02 | 0.59 |
| 2019 | 0.81 | 0.17 | 0.70 | 0.13 | 0.76 | 0.31 | 0.96 | 0.29 | 0.85 | 0.25 | 0.91 | 0.54 |
| 2020 | 0.62 | 0.13 | 0.59 | 0.12 | 0.61 | 0.25 | 0.74 | 0.24 | 0.77 | 0.23 | 0.76 | 0.47 |
| 2021 | 0.50 | 0.11 | 0.61 | 0.12 | 0.55 | 0.24 | 0.75 | 0.26 | 0.64 | 0.20 | 0.70 | 0.46 |

CDR: Crude death rate; SM: Standardized mortality

**Table** **S4 Sensitivity analysis with intervention onset shifted to 2011**

|  | *β* | Newey–West  *SE* | *t* | *P* | 95%*CI* |
| --- | --- | --- | --- | --- | --- |
| *β_0_* | -0.106 | 0.054 | -1.97 | 0.066 | (-0.221~0.008) |
| time(*β_1_*) | 0.007 | 0.011 | 0.68 | 0.503 | (-0.015~0.030) |
| intervention(*β_2_*) | 0.031 | 0.115 | 0.27 | 0.789 | (-0.213~0.275) |
| time_after_intervention(*β_3_*) | -0.081 | 0.013 | -6.30 | <0.001 | (-0.108~-0.054) |

Newey-West SE : heteroskedasticity- and autocorrelation-consistent standard error (Newey-West, lag=2). Outcome variable is the natural logarithm of standardized rural CHD mortality.

**Table** **S5 Sensitivity analysis with intervention onset shifted to 2012**

|  | *β* | Newey–West  *SE* | *t* | *P* | 95%*CI* |
| --- | --- | --- | --- | --- | --- |
| *β_0_* | -0.080 | 0.046 | -1.75 | 0.100 | (-0.177~0.017) |
| time(*β_1_*) | -0.003 | 0.009 | -0.30 | 0.769 | (-0.021~0.016) |
| intervention(*β_2_*) | 0.109 | 0.048 | 2.26 | 0.038 | (0.007~0.211) |
| time_after_intervention(*β_3_*) | -0.085 | 0.011 | -7.89 | <0.001 | (-0.108~-0.062) |

Newey-West SE : heteroskedasticity- and autocorrelation-consistent standard error (Newey-West, lag=2). Outcome variable is the natural logarithm of standardized rural CHD mortality.
